# A computational study on the influence of insect wing geometry on bee flight mechanics

**DOI:** 10.1242/bio.024612

**Published:** 2017-10-23

**Authors:** Jeffrey Feaster, Francine Battaglia, Javid Bayandor

**Affiliations:** Department of Mechanical and Aerospace Engineering, University at Buffalo, Buffalo, NY 14260, USA

**Keywords:** Insect flight, Wing cross-section, Bee, Aerodynamics, Morphological accuracy

## Abstract

Two-dimensional computational fluid dynamics (CFD) is applied to better understand the effects of wing cross-sectional morphology on flow field and force production. This study investigates the influence of wing cross-section on insect scale flapping flight performance, for the first time, using a morphologically representative model of a bee (*Bombus pensylvanicus*) wing. The bee wing cross-section was determined using a micro-computed tomography scanner. The results of the bee wing are compared with flat and elliptical cross-sections, representative of those used in modern literature, to determine the impact of profile variation on aerodynamic performance. The flow field surrounding each cross-section and the resulting forces are resolved using CFD for a flight speed range of 1 to 5 m/s. A significant variation in vortex formation is found when comparing the ellipse and flat plate with the true bee wing. During the upstroke, the bee and approximate wing cross-sections have a much shorter wake structure than the flat plate or ellipse. During the downstroke, the flat plate and elliptical cross-sections generate a single leading edge vortex, while the approximate and bee wings generate numerous, smaller structures that are shed throughout the stroke. Comparing the instantaneous aerodynamic forces on the wing, the ellipse and flat plate sections deviate progressively with velocity from the true bee wing. Based on the present findings, a simplified cross-section of an insect wing can misrepresent the flow field and force production. We present the first aerodynamic study using a true insect wing cross-section and show that the wing corrugation increases the leading edge vortex formation frequency for a given set of kinematics.

## Abstract (precis)

**Summary:** A morphologically accurate insect wing cross-section is compared with common cross-sections reported in the literature. It was found that misrepresenting the insect wing cross-section can result in different aerodynamics. This article has an associated First Person interview with the first author of the paper as part of the supplementary information.

## INTRODUCTION

Biological flapping flight is an extraordinarily complex phenomenon, which occurs across multiple phylums of the animal kingdom, with wide disparities in the balance between aerodynamic performance and efficiency (Ellington, 1985; Walker et al., 2014; Mao and Gang, 2003; Miyake, 2008). The powered flight of insects is inherently unique for its combination of mechanical simplicity and high flapping frequency, resulting in a complicated vortical flow field. These flight characteristics result in agile and maneuverable flight capabilities superior to fixed wing flight such as aircraft (Taylor et al., 2003). Bees are of particular interest because of the utilization of humuli to attach their front and hind wings together during flight, causing the front and hind wings to move as one body (Basibuyuk and Quicke, 1997). Additionally, a bee can carry an additional 80% of its own body weight for miles (Winston, 1991). Bee aerodynamics is uniquely applicable to the future direction of micro-air vehicle (MAV) research as another possible solution to low velocity, low Reynolds number, large payload flight (Ma et al., 2012; Yan et al., 2001; Bai et al., 2007).

To support the creation of more effective MAV systems, it is critical that the current understanding of insect flapping flight is expanded. The first modern explorations into the fluid dynamics of insect flight were experiments utilizing particle image velocimetry (PIV) and other visualization methods (Ellington, 1984; Casey et al., 1985; Mazaheri and Ebrahimi, 2010; Carr and Ringuette, 2014). An experiment by Morse and Liburdy (2009) at low Reynolds number characterized the vortex field around a static wing at high angles of attack for flow over a flat plate. Insect flight is progressively becoming a focus area for fluid and structural researchers alike as computational capabilities improve. The dual improvement of numeric modeling methodologies and computing power is allowing for progressively more accurate and refined computational models of flapping flight (Hu and Xiao, 2014; Nakata and Liu, 2012; Aono et al., 2008). The advances in computational modeling and resulting aerodynamic understanding of complex flight phenomenon is directly aiding the development of robotic MAV systems (Wood et al., 2013). There have been multiple attempts to create low-order models approximating more complex computational fluid dynamics (CFD) results, which could be implemented into various control schemes (Amiralaei et al., 2012; Singh et al., 2004; Taha et al., 2014; Bayandor et al., 2013; Dadashi et al., 2016).

Numerous experimental and computational studies have investigated insect flapping flight (for a thorough review of these topics see Ansari et al., 2006; Saha and Celata, 2011; Shyy et al., 2010). These investigations focused on one of four insect flight strategies: fruit flies (*Drosophila*), which utilize a single pair of wings to flap at high frequency; dragonflies (*Sympetrum*), which move four wings independently at high frequency; hawkmoths (*Sphingidae*), which utilize four wings flapping in synchrony at low frequency; and bees (*Apoidea*), which utilize four wings flapping at high frequency (Miyake, 2008; Shyy et al., 2010).

Three-dimensional (3D) CFD investigations into insect flight have been performed by numerous groups. Ramamurti and Sandberg (2002) computationally investigated the unsteady flow over a fruit fly and successfully compared it to the experimental results quantified by Dickinson et al. (1999). Additional 3D CFD investigations of a fruit fly have been performed by Walker (2002) utilizing an unsteady blade element model, and Aono et al. (Aono and Liu, 2008; Aono et al., 2008) utilizing an incompressible, unsteady form of the Navier-Stokes equations. The hovering and forward flight of a wide range of insects, including bees, have been analyzed three-dimensionally for both force production and aerodynamic efficiency by Du and Sun (2008, 2012). Du and Sun also reported that a simplified form of the naturally occurring wing corrugation had minimal impact when compared to a flat plate undergoing the same kinematics (Du and Sun, 2012). In addition to the previously discussed 3D simulations, numerous two-dimensional (2D), unsteady computational analyses have been performed by various groups. Wang (Wang, 2000; Wang et al., 2004) studied hoverfly hovering, Lu and Liao (2006) explored the effects of vertical undulation of a rigid ellipse, Ishihara et al. (2009) evaluated the characteristics of passive pitching of a hoverfly undergoing hovering and Kolomenskiy et al. (2011) computationally modeled the differences between 2D and 3D for a generalized flapping system. In addition to their respective insights, each group found good agreement with 3D computational and experimental models of various flapping flight phenomenon.

At present, of the papers directly dealing with computational or experimental studies on insect flight mechanics published since 1998, five papers implement a triangular corrugated cross-section (Meng and Sun, 2011; Du and Sun, 2012; Khurana and Chahl, 2014; New et al., 2014), six papers have used a NACA0012 airfoil (Amiralaei et al., 2009; Chandar and Damodaran, 2008, 2010; Amiralaei et al., 2010; Tian et al., 2014), 10 use an elliptic cross-section (Wang, 2000; Mao and Gang, 2003; Lu and Liao, 2006; Berman and Wang, 2007; Kim and Choi, 2007; Hamdani and Naqvi, 2011; Amiralaei et al., 2011; Zhu and Zhou, 2014; Hu and Xiao, 2014; Du and Sun, 2015), and 23 utilize a flat plate (Liu et al., 1998; Ramamurti and Sandberg, 2002; Sun and Lan, 2004; Wang et al., 2004; Wu and Shao, 2004; Saputra et al., 2006; Huang et al., 2007; Zuo et al., 2007; Aono et al., 2008; Aono and Liu, 2008; Liu, 2009; Ishihara et al., 2009; Senda et al., 2010; Yin and Luo, 2010; Sudhakar and Vengadesan, 2010; Kolomenskiy et al., 2011; Lee et al., 2008, 2011; Nakata et al., 2011; Nakata and Liu, 2012; Ishihara et al., 2014; Shen and Sun, 2015; Erzincanli and Sahin, 2015). Of these papers, seven (Zuo et al., 2007; Meng and Sun, 2011; Luo and Sun, 2005; Mao and Gang, 2003; Berman and Wang, 2007; Liu, 2009; Wu and Sun, 2005) investigate the aerodynamics associated with bee aerodynamics in either hovering or forward flight. Although the aerodynamic effects of a basic corrugation have been determined to be negligible (Meng and Sun, 2011; Meng et al., 2011), the effects of more extreme corrugation associated with biologically accurate wing cross-sections remain unexplored.

To this end, the aerodynamic effects of cross-sectional geometry for a bee over the natural velocity range of bee flight is studied numerically using the unsteady, incompressible form of the Navier-Stokes equations in two dimensions. The goals of the present study are twofold. The first is to determine if misrepresentation of wing cross-section in insect flight adversely influences observed force and flow phenomenon. The second is to determine how the results of commonly used cross-sections compare with those using morphologically accurate venation. Transient and average coefficients of lift and drag are compared in depth to improve the understanding of the possible aerodynamic differences due to wing cross-section. The wing kinematics are based on high speed videography by Ellington and Dudley (1990) and are used as a basis for equations of motion to approximate the insect wing path. This work is the first computational, morphologically accurate analysis of a bee wing cross-section with direct comparisons to common cross-sectional geometries presented in the literature. The underlying fluid mechanics caused by the wing cross-sectional geometry can manifest in extreme variations in thrust, lift, and vortex development for different cross-sections undergoing the same kinematics. The results presented will determine whether the complexity of a bee wing cross-section can be simplified for further modeling and will establish the necessity of morphologically accurate models of wing geometry to predict representative aerodynamic forces for bee flight, and further, may yield additional passive methods to control vortex formation during flight that have not been studied previously.

## RESULTS

### Flow characteristics

**Fig. 1.**
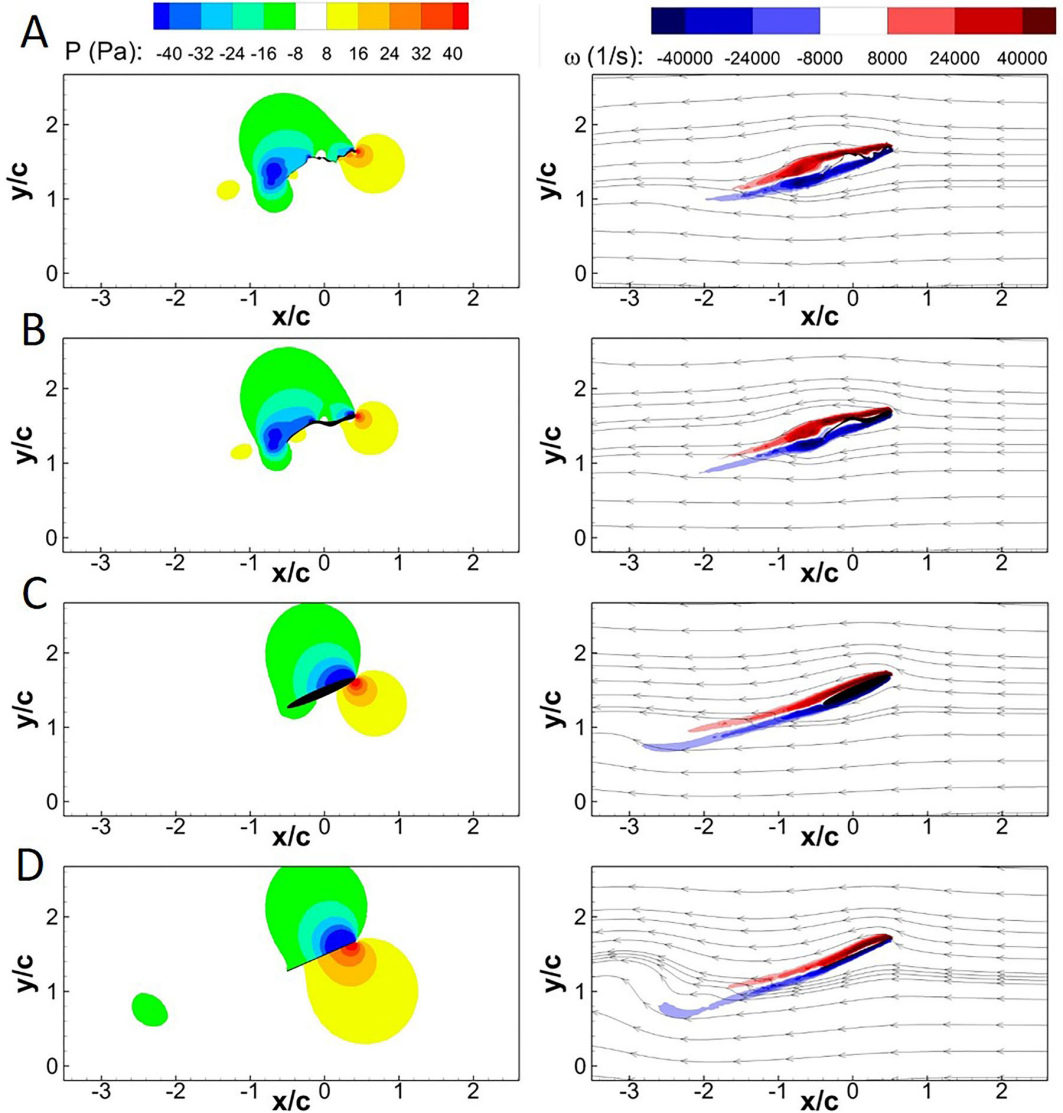
**Pressure and vorticity contours at *τ*=11.25 for the four wing cross-sections at 5 m/s.** Pressure (left column) and streamlines with vorticity (right column) contours at *τ*=11.25 are shown in the (A) bee, (B) approximated, (C) ellipse and (D) flat plate cross-sections.

**Fig. 2.**
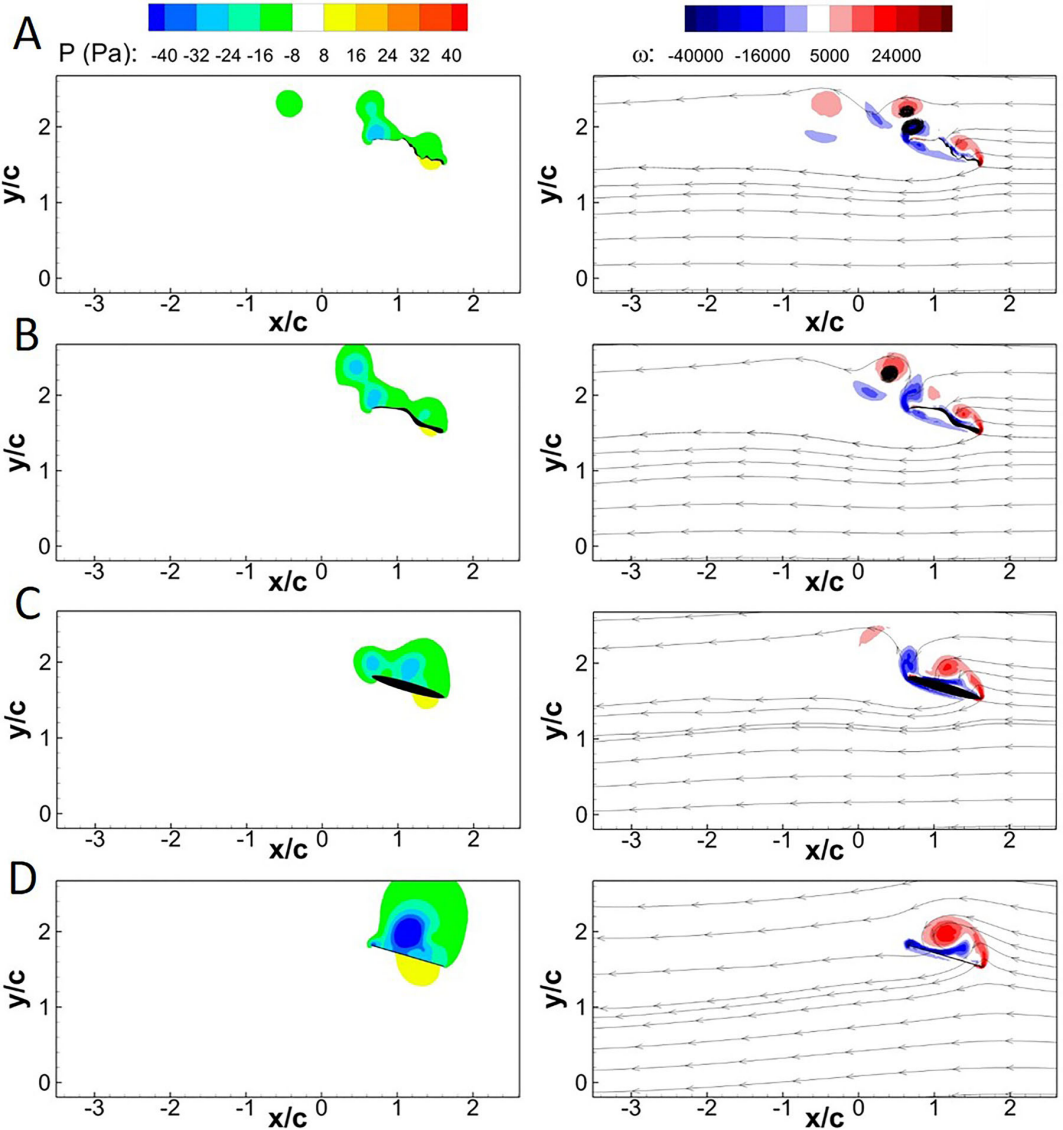
**Pressure and vorticity contours at *τ*=11.68 for the four wing cross-sections at 5 m/s.** Pressure (left column) and streamlines with vorticity (right column) contours at *τ*=11.68 are shown in the (A) bee, (B) approximated, (C) ellipse and (D) flat plate cross-sections.

**Fig. 3.**
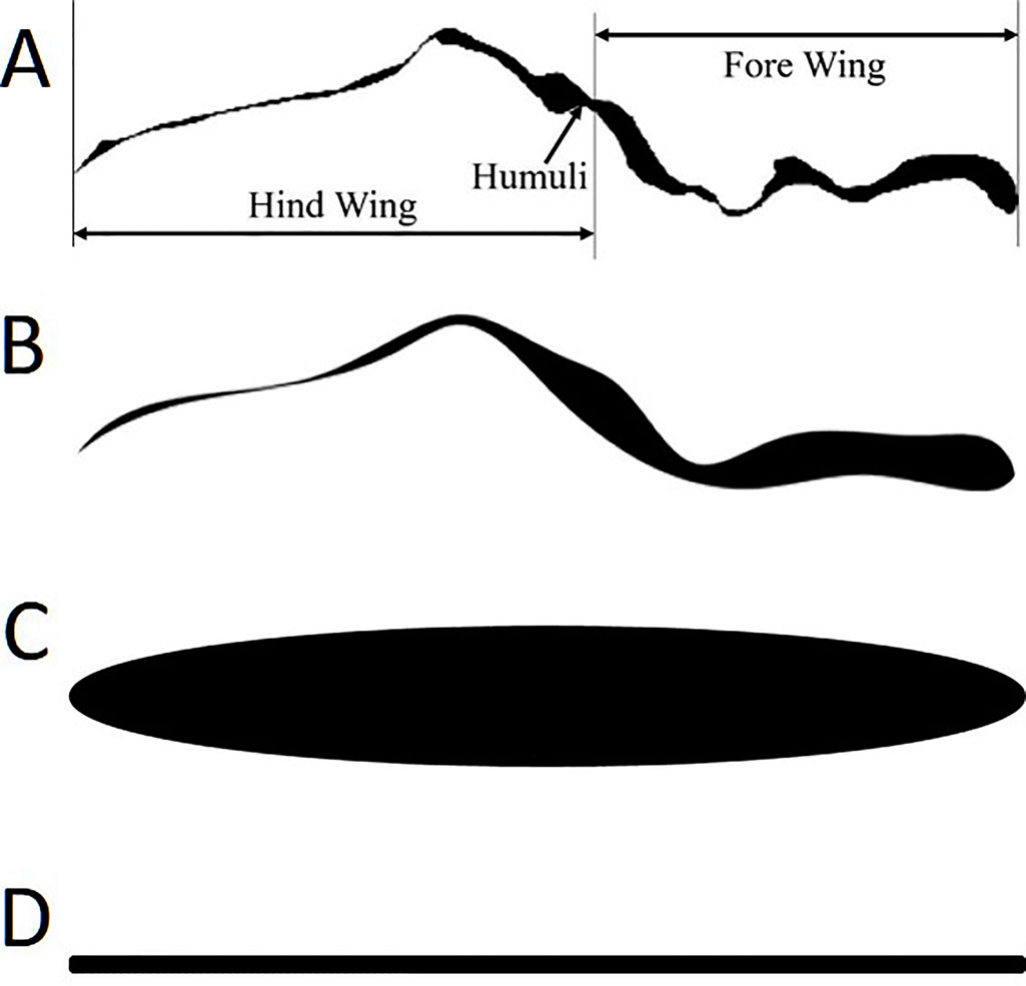
**The cross-sections used in the present analysis.** (A) Bee, (B) approximated, (C) ellipse and (D) flat plate.

The flow fields at *τ*=11.25 (*τ*=0.25 in Fig. S2) and 11.68 (between *τ*=0.5 and *τ*=0.75 in Fig. S2) are shown in Figs 1 and 2, respectively, to gain insight into the underlying aerodynamics that manifests as differences in lift and drag. The parameter tau is normalized by the flap period. Parts A and B of both figures show the pressure and vorticity distributions with streamlines, respectively, in a portion of the flow field directly around the wing. The wing cross-sections are organized in descending order from most (bee cross-section) to least geometrically complex (flat plate), as shown in Fig. 3.

Beginning with Fig. 1 (*τ*=11.25), a leading edge vortex (LEV) is already in the process of shedding from the hind wing section (lowest pressures), while the front bee wing section shows the initial formation of an LEV for both the bee and approximated wing cross-sections. The trailing edge vortex (TEV) is almost fully detached from the bee cross-section, visible in the location of the low pressure region on the upper portion of the wing, with distinct lack of vorticity in the same region. The LEV on the approximated wing section is still in the process of detaching from the wing. The bee cross-section sheds the hind wing LEV earlier in the stroke than the approximate section. The postponement of vortical shedding increases the area and pressure gradient of the vortex for the approximate cross-section, increasing lift generation. Both the ellipse and flat plate cross-sections are still in the process of forming an LEV on the front of the wing. The underside of the ellipse wing exhibits a small low pressure region near the trailing edge, transitioning to a high pressure region towards the leading edge. The transition from high to low pressure is similar to what is seen in the bee and approximated sections, where the pressure transitions from high to low to high, with the high pressure regions caused by stagnation points on the cross-section. Conversely, the flat plate section has low pressure on the upper and high pressure on the lower surfaces of the wing. These variations in pressure are caused by differences in the vortex generation and shedding capabilities of the cross-sectional geometry of the wing.

Examining Fig. 2, the flow field surrounding the four cross-sections are compared at *τ*=11.68. The bee and approximate cross-sections both exhibit multiple vortices shed from both the leading and trailing edges. The bee and approximate wing cross-sections have two attached vortex structures (LEV and TEV) on the upper portion of the wing. The bee cross-section exhibits a second set of vortices located further downstream which are not present in the approximate section. The ellipse and flat plate sections show progressively differing flow structures. The ellipse cross-section exhibits attached TEV and LEV similar to those seen in the approximated and bee sections, with a higher vorticity magnitude. The ellipse cross-section only shows a single remnant LEV entrained in the flow. Although the number of vortex structures immediately surrounding the ellipse are similar to those seen by the approximate and bee cross-sections, the overall locations of the vortices are different. The difference in vortex locations relative to the wing itself drastically changes the distribution of pressures along the wing, moving the low pressure region associated with the LEV towards the cross-sectional center. Comparing the bee, approximate and ellipse cross-sections with the flat plate, there is a distinct reduction observed in flow complexity with the flat plate, which generates a large single LEV.

## DISCUSSION

The goal of the present study is to establish the aerodynamic differences among wing cross-sections by comparing biologically accurate wing sections with cross-sections commonly used in the computational literature. In order to establish the effects of biologically accurate wing cross-section on bee flight aerodynamics, the wing kinematics outlined in section are applied to all four cross-sections shown in Fig. 3. The induced fluid mechanics are compared using vorticity and pressure contours with the resulting forces compared from both an instantaneous and time averaged perspective.

Owing to the identical nature of the kinematics, differences in the flow field and resulting forces will be considered to be caused by the cross-sectional variation. Throughout the discussion, *τ* will be used to define the phase of the wing flap stroke. The discussion will refer to a given phase after a number of flap cycles as *τ*=11.25, where the solution is 0.25 *τ* after 11 strokes. The relative position of *τ* is shown in Fig. S2.

### Variations in lift coefficient

**Fig. 4.**
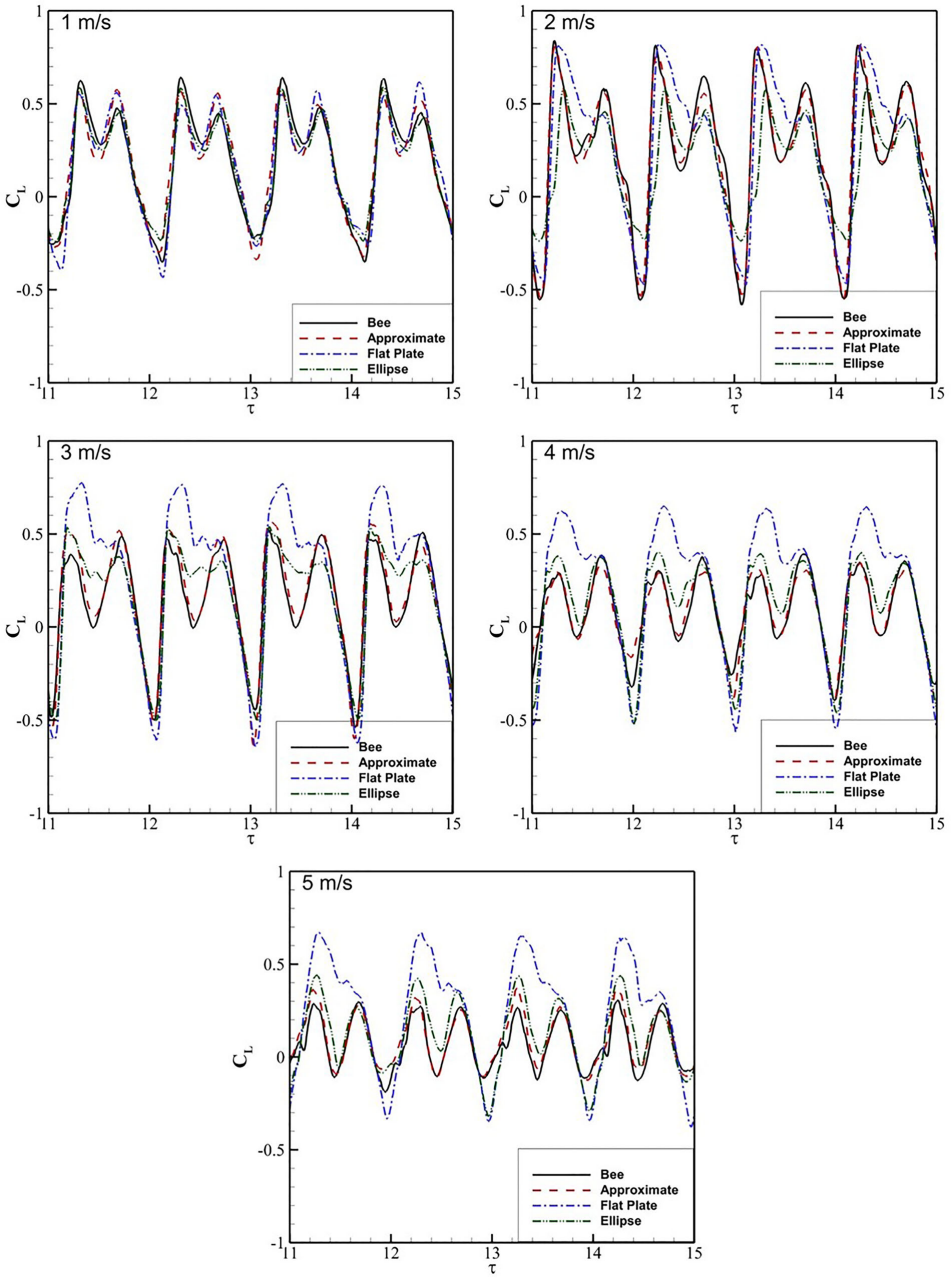
**The time history of *C_L_* for the bee, approximated, elliptic and plate cross sections are shown over four flap periods**. Each graph is associated with a different flow velocity beginning at 1 m/s at top left and ending at 5 m/s at the bottom.

Fig. 4 presents the *C*_*L*_ data for four flap cycles comparing the flat plate, ellipse, approximate and bee wing cross-sections at *τ* at *V*_∞_=1−5 m/s. At all speeds, the bee and approximate wings exhibit two distinct peaks in *C*_*L*_, which correlate with LEV shedding at approximately *τ*=11.75 (the midpoint of the upstroke) and TEV shedding at *τ*=11.75 (midpoint of the downstroke). At 1 m/s, all cross-sections produce a very similar transient *C*_*L*_. At 2 m/s the flow field of the flat plate begins to differ from the other three cross-sections such that the LEV structure does not shed at the stroke transition, causing a more gradual drop in lift production. The difference in aerodynamic forces become more notable at 3 m/s where the flat plate and, to a lesser degree, the ellipse do not exhibit the reduction in *C*_*L*_ at *τ*=11.5. As *V*_∞_ increases to 5 m/s, the elliptical wing profile predicts similar *C*_*L*_ to the bee and approximate wings. Meanwhile the flat plate profile continues to produce very high lift, more than double the peak force experienced by the bee cross-section. The trends at 4 m/s remain consistent with those observed at 3 m/s, with a progressively different shedding pattern as wing cross-sectional geometry differs from the biological model. At 5 m/s, *C*_*L*_ for the bee wing and the approximate wing remains in good agreement. The peak *C*_*L*_ is substantially higher for the flat plate and the minimum *C*_*L*_ is significantly lower for both the ellipse and flat plate cross-sections. The difference in lift generation indicates that a simplified wing cross-sections will not necessarily yield biologically representative *C*_*L*_.

Comparing time-averaged *C*_*L*_, in Table 1, all cross-sections except for the elliptic one exhibit a significant increase in *C*_*L*_ at 2 m/s. After 2 m/s, the *C*_*L*_ for the bee, approximate and ellipse cross-sections decrease steadily, with the exception of 5 m/s for the approximate cross-section, which increases to 0.1. The flat plate exhibits a consistent *C*_*L*_ from 2 to 5 m/s, while the other cross-sections display a progressive decrease. The approximate cross-section *C*_*L*_ is consistently closest to the bee results, with a maximum difference of 0.03.

**Table 1.**
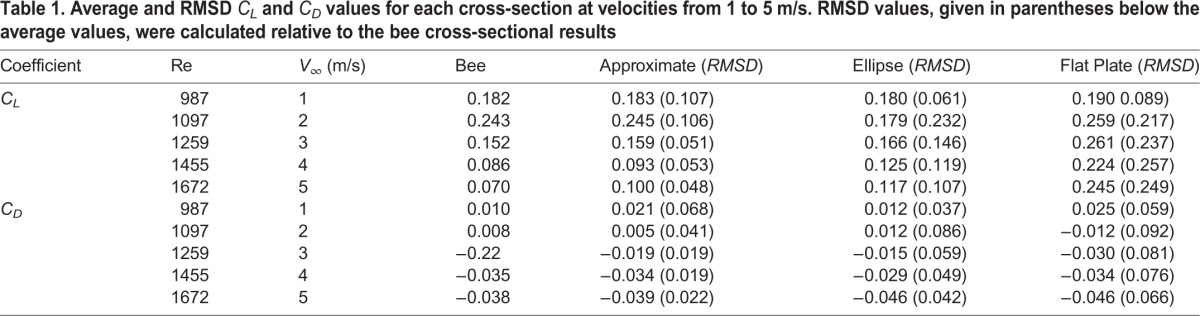
**Average and RMSD** *C*_*L*_ **and** *C*_*D*_ **values for each cross-section at velocities from 1 to 5 m/s. RMSD values, given in parentheses below the average values, were calculated relative to the bee cross-sectional results**

The root mean squared difference (RMSD) will be used to quantify the transient fluctuations from the biologically accurate cross-section present in the instantaneous data. Investigating both time-averaged force results and RMSD allows for the quantification of both instantaneous and average error.

The *C*_*L*_ root mean square difference 
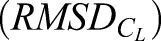
 for each of the cross-sections is compared to the bee, where the 
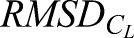
 is calculated as
(1)
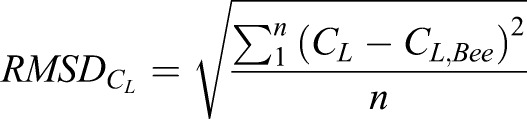
and *n* is the number of time steps analyzed over 1600 time steps from *τ*=3–15. The 
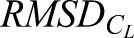
 is proportional to the instantaneous difference in *C*_*L*_ from the bee cross-section; the larger the value, the more disparate the instantaneous forces. Table 1 shows the 
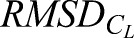
 of each of the cross-sections relative to the bee. At 1 m/s the ellipse *RMSD*_*CL*_ was the closest to the bee. Once the velocity increases to 2 m/s, the ellipse and flat plate average *C*_*L*_ begin to differ significantly from the bee results while the 
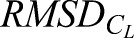
 remains consistent. Increasing velocity from 2 m/s to 3 m/s, the 
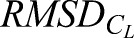
 of the approximate cross-section decreases by ∼50% while the flat plate remains consistently high. For velocities between 3 and 5 m/s, the 
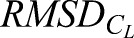
 for each of the cross-sections remains the same with the approximate section consistently exhibiting the closest similarity, followed by the ellipse and flat plate cross-sections.

### Variations in drag coefficient

**Fig. 5.**
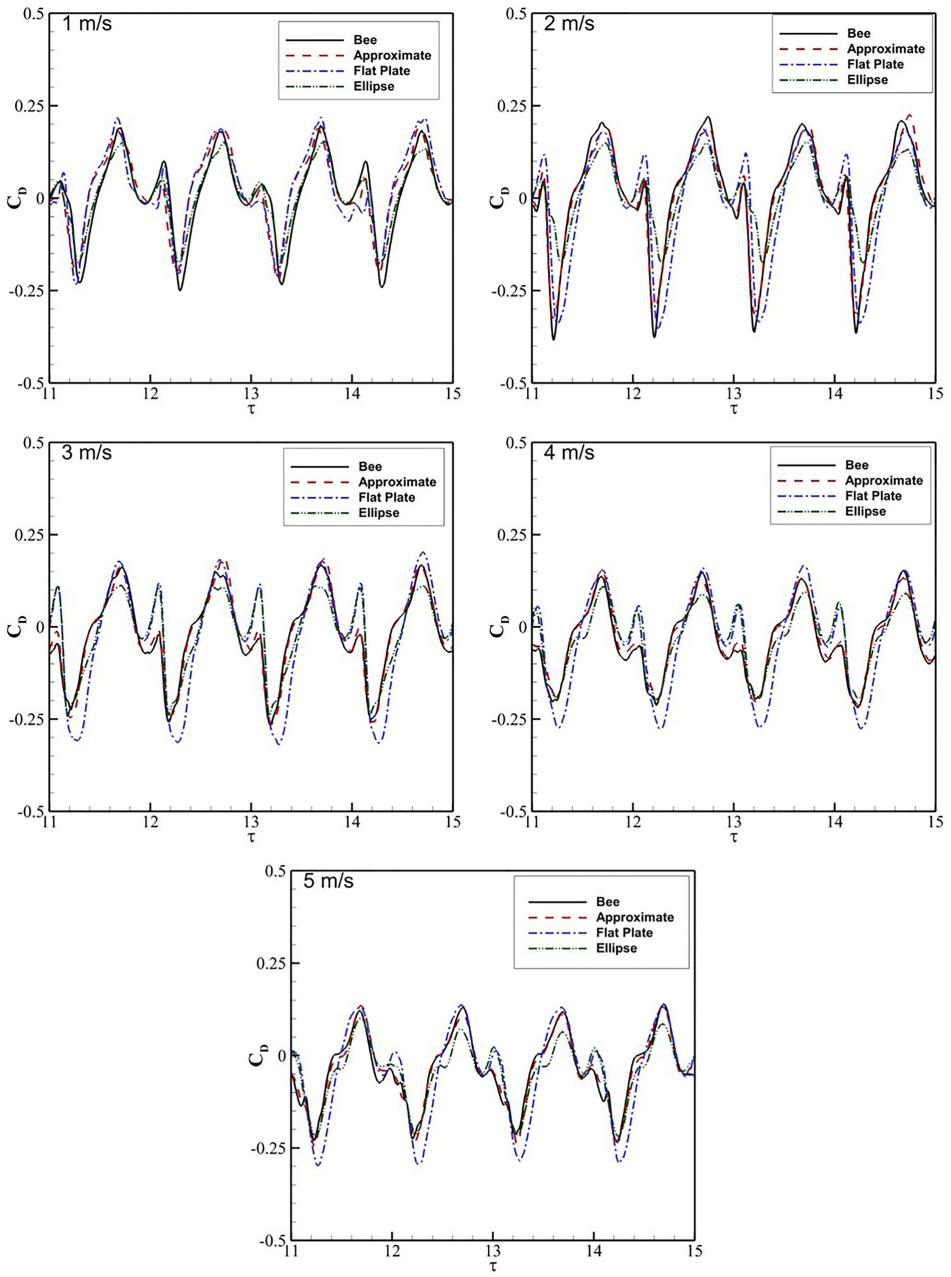
**The time history of *C_D_* for the bee, approximated, elliptic and plate cross sections are shown over four flap periods**. Each graph is associated with a different flow velocity beginning at 1 m/s at top left and ending at 5 m/s at the bottom.

Fig. 5 presents the *C*_*D*_ data for four flap cycles comparing each wing cross-section versus *τ* at *V*_∞_=1−5 m/s. The *C*_*D*_ values at 1 m/s agree well between all four cross-sections. Each cross-section exhibits an initial peak at the start of the upstroke, before dropping to the minimum *C*_*D*_ at *τ*=11.2. After *τ*=11.2, *C*_*D*_ increases steadily to the maximum around *τ*=11.7. After the maximum at *τ*=11.7, there is another drop in *C*_*D*_ at *τ*=11.9. From *τ*=11.9 to *τ*=12, *C*_*D*_ increases again, peaking immediately after transitioning back to the upstroke.

At 3 m/s, the *C*_*D*_ of the ellipse and flat plate cross-sections begin to shift from the approximate and bee wings. All four cross-sections still follow the same *C*_*D*_ trends discussed with 1 m/s, with a number of minor variations. The peak at *τ*=11 is significantly higher for both the flat plate and ellipse sections, which agree well with one another, unlike the *C*_*D*_ for the approximate and true bee wing. The minimum *C*_*D*_ at *τ*=0.2 is significantly lower for the flat plate with a phase offset of 0.1*τ*, while the minima for the ellipse, approximate and flat plate agree well in both phase and magnitude.

Comparing the time-averaged *C*_*D*_ values in Table 1, the differences in average *C*_*D*_ are small between the four cross-sections for all of the flight velocities. The airfoils develop thrust at 1 and 2 m/s, but experience a net drag at higher velocities. The approximate section is closest to the bee *C*_*D*_ for all simulations except 1 m/s. At 2 m/s the flat plate cross-section transitions from generating thrust to net drag production slightly sooner than the other cross-sections. The *C*_*D*_ root mean square difference 
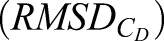
 for each of the cross-sections is compared to the bee, where the 
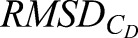
 is calculated as
(2)
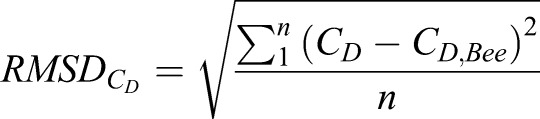
The 
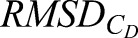
 for each cross-section relative to the bee is shown in Table 1. The ellipse section had the lowest 
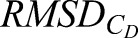
 at 1 m/s. Increasing the velocity to 2 m/s, the approximate section increases in accuracy while the elliptical and flat plate decrease, counter to what was seen with 
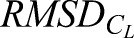
. From 3 to 5 m/s, the 
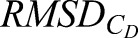
 remains relatively consistent for the approximate wing and decreases consistently for the ellipse and flat plate. At 5 m/s, the approximate cross-section remains the closest to the bee, with the ellipse and flat plate being roughly two and three times the approximate 
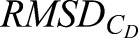
, respectively.

The similarity in the vorticity and pressure contours manifest in the similarities in *C*_*L*_ and *C*_*D*_ as previously discussed, both average and *RMSD*. Evidenced by both instantaneous forces and the flow field in Fig. 1, the wing cross-sectional complexity coincides with distinctive changes in vortical shedding, pressure distribution over the wing, and force production at *τ*=11.25. Conversely, while the four cross-sections exhibit very similar instantaneous *C*_*L*_ and *C*_*D*_ at *τ*=11.68, it is clear that there can still be large disparities in the respective flow fields evidenced by the extreme variation in pressure and vorticity distribution at that instant.

The findings presented demonstrate the effects of wing profile on both the immediate flow field and the resulting aerodynamics. The approximated wing profile that represents a streamlined bee cross-section produced the most consistent similarity with the results of the bee cross-section across all metrics. The ellipse profile was the second best at representing the aerodynamics associated with the bee cross-section, capturing the underlying physics more consistently than the flat plate. However at low velocities, the profile thickness of the ellipse changed the underlying vortex dynamics. The flat plate generally captured the aerodynamics associated with the bee-profile the least, with a large LEV consistently forming and not detaching until well into the downstroke. The large LEV formation and shedding experienced by both the flat plate and ellipse, caused by the transition from the up to the downstroke (*τ*=0.5), does not fully shed from the wing until *τ*=0.6-0.7. This is earlier than the vortex structure shedding for the bee and approximate cross-sections, which is much closer to *τ*=0.5. The large LEV structure creates a larger net lift, consistently increasing both the average and 
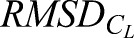
. Finally, the present study shows that neglecting the intricacies of a highly complex wing geometry can produce results that, while physical, do not properly capture the surrounding flow field or resulting forces experienced by the biological system. The difference in performance of the bee versus the approximated, ellipse and flat plate wing cross-sections highlights the importance of high resolution modeling of insect wings, both to properly capture the underlying physics using CFD, and to maximize the effectiveness of insect-based MAV applications.

### Summary

The influence of wing cross-section on aerodynamic performance was investigated using flight velocities and flapping kinematics for a bee. Four different representations of an insect wing cross-section were compared using the cross-section from a micro-computed tomography scan of an actual bee wing and an approximation of that bee wing cross-section. Two other cross-sections were also compared based on the most prevalent in the literature: an ellipse, the second most prevalent, and a flat plate cross-section, the most prevalent. Each wing cross-section was simulated using the same mesh density, flight speed, and kinematics. Each wing profile was compared using the surrounding flow field, instantaneous *C*_*L*_ and *C*_*D*_, time-averaged *C*_*L*_ and *C*_*D*_, and RMSD of *C*_*L*_ and *C*_*D*_ for each cross-section to gain a more quantitative representation of the instantaneous variation in *C*_*D*_ and *C*_*L*_.

Qualitatively, these kinematic motions exhibited similar vortex patterns among the four cross-sections, generating LEV-TEV pairs. Vortex formation differed significantly in the number and timing of shedding for each cross-section causing distinctly different resulting forces. The bee and approximate cross-sections were generally comparable both qualitatively and quantitatively to one another. Vortex shedding complexity was shown to increase with wing cross-section complexity, with the flat plate and elliptical cross-sections often forming a single LEV structure over a flap period while the more morphologically accurate model shedded two distinct LEV structures for the same kinematics. With regards to *C*_*D*_, net thrust was produced for all wings at low velocities before transitioning to net drag production at higher velocities. The present study has shown that cross-sectional morphology can significantly influence the occurrence and frequency of LEV formation, changing observable flow structures and aerodynamic performance. This change in vortex formation and shedding frequency augments the effects of dynamic stall, a major source of lift production for biological flight. Based on the present findings, the influence of wing cross-section on aerodynamic performance cannot be assumed to be consistently negligible at the insect scale for future computational analysis and MAV applications, and should be evaluated on a case-by-case basis, with better understanding of cross-sectional effects yielding an additional passive mechanism for LEV formation control.

## MATERIALS AND METHODS

### Governing equations

The commercial software ANSYS Fluent (v. 15) is used to solve the velocity and pressure fields around a dynamic 2D wing cross-section. The unsteady, incompressible Navier-Stokes equations are employed, yielding the continuity equation
(3)
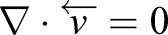
where *t* is time, 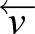 is the velocity vector and *ρ* is the fluid density. Neglecting gravitational effects, the momentum equations are
(4)
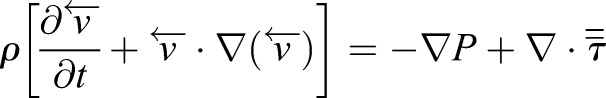
where *P* is the pressure and 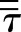 is the fluid stress tensor.

The segregated pressure-based Navier-Stokes (PBNS) solver is used to solve the incompressible flow. The SIMPLE algorithm is used to resolve the pressure-velocity coupling by enforcing mass conservation using a relationship between element pressure and flux corrections (ANSYS Incorporated, 2014). The gradients are discretized using the least squares cell based (LSCB) method, momentum equations are discretized using the second-order upwind scheme, and time is discretized using a first-order implicit method. The absolute convergence criteria for the solution variables are set to 10^–7^. The time step is determined using a modified version of the Courant-Fredrichs-Levy (CFL) number to suit the implementation of kinematic motion:
(5)
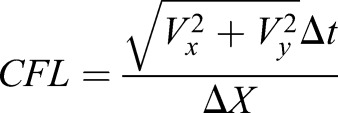
where Δ*t* is the time step, Δ*X* is the smallest cell size, and *V*_*x*_ *V*_*y*_ are the respective kinematic velocities in the *x*- and *y*- directions. The maximum CFL value used in the present simulations is 1 to ensure a stable calculation.

### Dynamic remeshing equations

An unstructured finite-volume mesh is generated around the cross-section and is updated with each time step to allow for proper mesh motion, using a combination of Laplacian smoothing and remeshing functions to retain mesh density around the wing at all points in time during the flapping motion. An unstructured, triangular 2D mesh is created around the wing profile (an example of the mesh immediately around the bee wing is shown in Fig. S1). Air enters the computational domain along the right boundary with a uniform velocity specified. The upper and lower boundaries of the fluid domain use a slip condition. The left side of the fluid domain is specified as ambient pressure (0 gauge). It should be noted that the entire domain is 88 mm×44 mm (22*c*×11*c*).

The diffusive smoothing function for the dynamic meshing is defined as
(6)
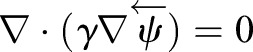
where the diffusion coefficient *γ* defines the degree to which the boundary motion 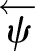 propagates through the surrounding fluid mesh. *γ* is calculated using the cell distance from the deforming boundary *d*:
(7)
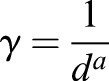
Solving Eqn 6 for 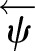, the mesh distribution at the next time step 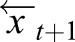 is solved using 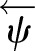, Δ*t* and the present mesh distribution 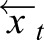:
(8)
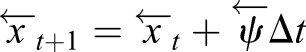
The diffusion parameter (*a*) was set to 1.75 for the study, and typically ranges between 0 and 2, where *a*=1 produces uniform diffusion and values greater than one cause regions further from the boundary to deform, while retaining resolution in the immediate vicinity of the moving boundary.

A remeshing function is implemented to resolve the extreme boundary motions present in flapping flight. A secondary background mesh is used to retain the consistency of the fluid data during remeshing. The remeshing function uses the nodal distance from the nearest boundary *d*_*min*_ and the most remote node from the boundaries *d*_*max*_ to normalize the boundary distance *d*_*b*_:
(9)
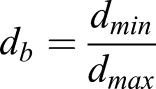
*d*_*b*_ is then used to determine the cell size at the location of interest (*size*_*i*_)
(10)
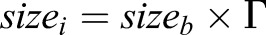
where Γ is the sizing function factor, defined by the size function variation *α* and the size function rate *β*. Two different equations for Γ are implemented depending on the sign of *α*:
(11)
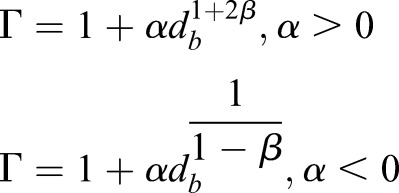
Combining Eqns 10 and 11 yields the allowable size of a cell in the fluid volume. Using the remeshing functions with diffusion smoothing creates a robust, highly refined mesh.

### Grid resolution study

The overall lift and drag coefficients for a wing profile are defined as
(12)
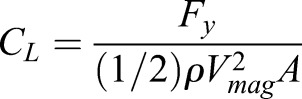

(13)
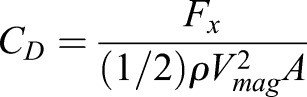
where *F*_*y*_=*L* is the lift force and *F*_*x*_=*T*−*D* is the force due to the difference between thrust *T* and drag *D*, *A* is the wing planform area, and *V*_*mag*_ is the maximum translational velocity magnitude of the wing. A positive *C*_*L*_ means that the wing is producing lift while a positive *C*_*D*_ represents the generation of thrust. Conversely, a negative *C*_*D*_ indicates a drag-dominated horizontal force.

The grid convergence index (GCI) methodology is used to determine the numerical accuracy of the solution using the difference between progressively refined meshes (Celik et al., 2008), where the number of cells *N* is used to quantify change in resolution. The grid resolution study is performed using a static mesh but with similar cell distributions as would be expected in the dynamic mesh simulation. Due to the nature of utilizing a static wing, *V*_*mag*_ is replaced with the inlet velocity *V*_∞_ for calculating Δ*t* using *CFL*. The grid resolution study examined steady-state lift and drag coefficients. The calculated order of accuracy of the simulations was found to be third order with a *C*_*D*_ at 506k yielding the maximum numeric error, 0.8%. Due to the variable nature of the mesh density when utilizing a remeshing scheme, the minimum resolution remained higher than 506*k* cells for all simulations presented herein (the full GCI results are given in Table S1).

### 2D validation

**Fig. 6.**
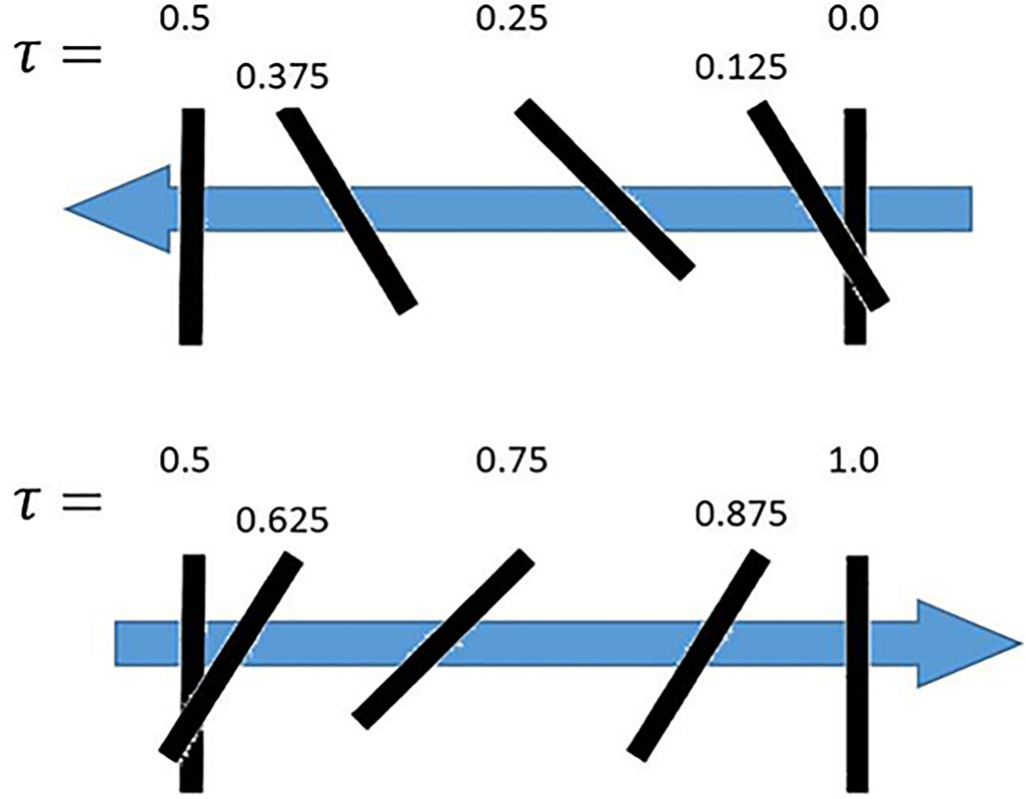
**Schematic of flat plate moving with validation kinematics over a full flap cycle.** The upstroke is shown at the top with an arrow signifying motion to the left. The motion reverses for the downstroke (at the bottom) with the wing translating to the right.

The CFD methodology is validated using digital particle image velocimetry (DPIV) experimental data reported by Wang et al. (2004) for flow over a hoverfly wing. The construction of the wing was such that the planform was the same shape as the hoverfly but with a uniform thickness. Thus, from a side view, the projected area is a simple rectangular cross-section. The 2D equivalent to the 3D geometry is analyzed as a flat plate with infinite depth in the CFD model. In the experiment, a robotic arm with three rotational degrees of freedom (DOF) and a flat Plexiglas wing with a hoverfly (*Drosophila*) planform was actuated in still mineral oil for Reynolds numbers between 50 and 200. The kinematics used in the in the validation are based on a fluid density of 880 kg/m^3^ and a kinematic viscosity of 1.15×10^–4^ m^2^/s. The kinematics used in the simulation are *f*=0.25 Hz, *r*=0.1625 m, *θ*_1*a*_=23.5^°^, *θ*_2*a*_=0^°^, *θ*_3*a*_=45^°^, φ_1_=*π*/2, φ_2_=0 and *c*=0.02385 m. All boundaries in the computational domain were modeled as no-slip walls to replicate the experimental tank. A schematic of the plate geometry orientation and kinematics are shown in Fig. 6. Fig. 6 and the remaining discussion utilize nondimensionalized time *τ*=*ft* to describe the location of the kinematics. For example, *τ*=4.25 coincides with the position *τ*=0.25 in Fig. 6 after four complete flap cycles. At *τ*=10 the flap cycle repeats, passing through the *τ*=0.125 location again.

**Fig. 7.**
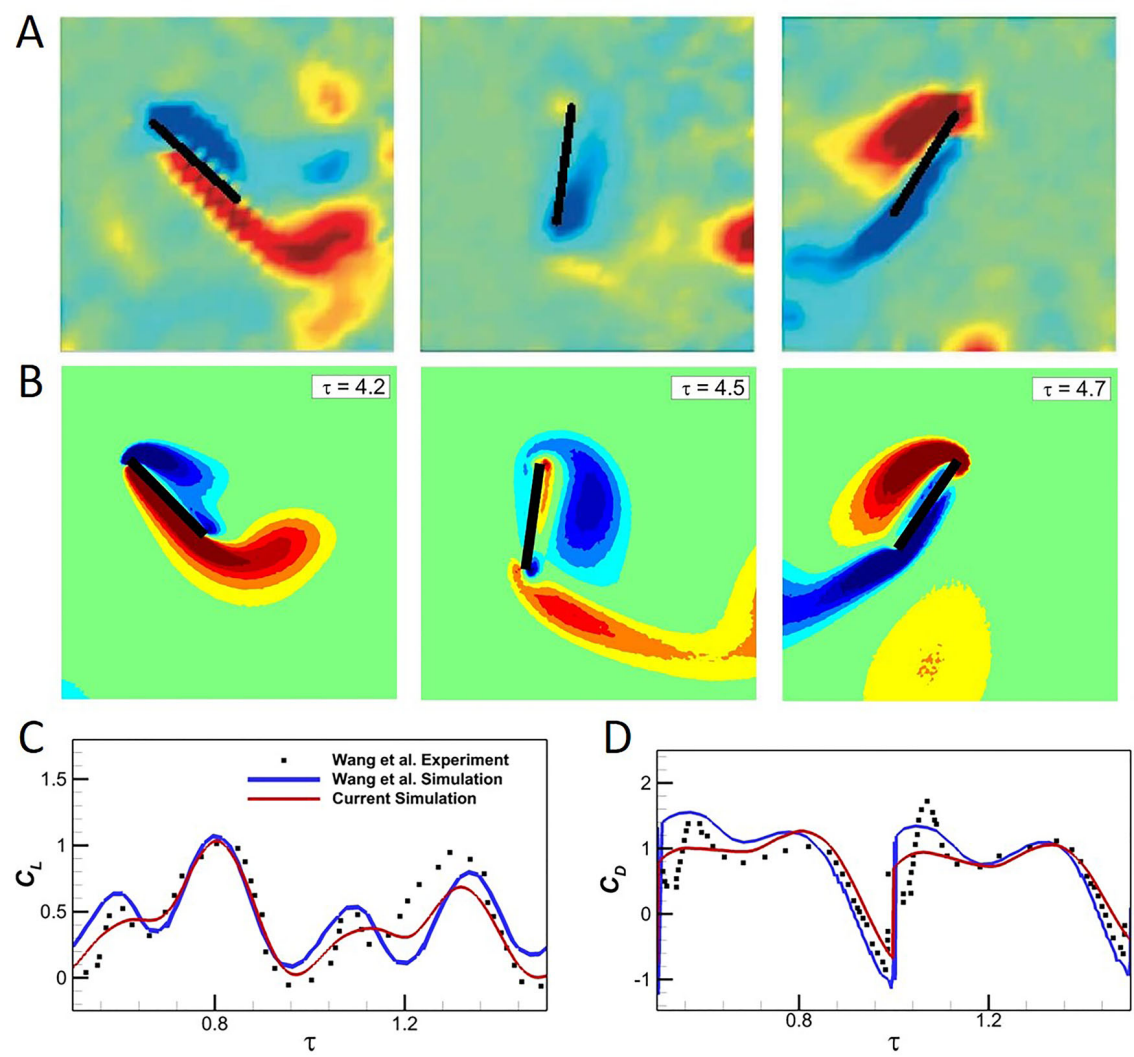
**Comparison of vorticities for the experimental and current simulations.** Qualitative comparison of vorticities for the (A) DPIV experimental (Wang et al., 2004) and (B) current simulations, where red signifies counter-clockwise and blue clockwise vortical rotation. The (C) *C*_*L*_ and (D) *C*_*D*_ versus *τ* for the present simulation methodology and the computational and experimental data of Wang et al. (2004) are shown.

Fig. 7 presents the DPIV data reported by Wang et al. (2004) (left) and the vorticity of the 2D simulations (right). The data at *τ*=4.2 (top), *τ*=4.5 (middle) and *τ*=4.7 (bottom) are compared and show good qualitative agreement between the simulations and the experiment with a very similar vortex shedding pattern. At *τ*=4.2, the wing is moving towards the left while rotating clockwise, causing a counter-clockwise vortex to form at the (lower) trailing edge and a clockwise vortex to form on the (upper) leading edge, which is captured in both the simulation and experiment. At *τ*=4.5, the wing starts moving to the right and is nearly vertical. The clockwise LEV that formed at *τ*=4.2 grows along the right side of the plate while the counter-clockwise TEV almost detaches. At *τ*=4.7 the wing moves further right, rotating clockwise while doing so. The motion causes a counter-clockwise vortex to form on the left edge of the plate and the LEV from *τ*=4.5 completely attaches.

In addition to the DPIV measurements, a 2D force sensor was attached to the base of the wing to capture the transient *C*_*L*_ and *C*_*D*_ of the wing (Wang et al., 2004). The transient simulation results shown in Fig. 7 exhibit close similarities to both the experimental and computational results reported by Wang et al. (2004). The largest differences occur at the stroke transition (e.g. *τ*=0.55 and 1.05). The variations at stroke transition are due to 3D effects in the experiment that are not captured in a 2D simulation. Quantitatively, the predicted time-averaged *C*_*L*_ and *C*_*D*_ values of 0.82 and 1.33 agree well with the experimental values given in Wang et al. (2004) of 0.86 and 1.34, respectively. The validation adds confidence to the 2D computational modeling to further pursue the wing profile analysis.

### Cross-sectional geometries

The cross-sections shown in Fig. 3 are those used in the remainder of the analysis. In descending order, the wings cross-sections are from a real bee, an approximation of the bee profile, an ellipse and a flat plate. The profiles in Fig. 3 are shown with the leading edge at the right and trailing edge at the left, with a chord length of *c*=4 mm.

The bee wing cross-section (Fig. 3A) used in the analysis is from a worker bee of the species *Bombus pensylvanicus*. The bee had died with wings fully extended, preserving the natural humuli connection between the front and hind wings. A Skyscan 1172 high resolution micro-computed tomography scanner with a resolution of 2 μm was used to generate a 3D model of the wing. The cross-section shown in Fig. 3 is taken at the midpoint of the wing between the wingtip and joint, capturing the fore and hind wing sections in addition to the humuli interface.

The approximate wing profile (3*b*) is based on the bee wing cross-section using the original point cloud of the bee wing, with manually smoothed vein structures. The removal of the vein structures and smoothing of the overall profile will help establish whether the irregular corrugation of the real wing dramatically affects the vorticity development. The ellipse (3*c*) and flat plate cross-sections (3*d*) are geometric representations commonly implemented in the literature instead of the biologically accurate insect cross-section. The elliptical cross-section has a maximum thickness of 0.125*c*, where thicknesses between 0.1*c* and 0.3*c* (Berman and Wang, 2007; Wu and Sun, 2005) were used in the bee-related literature. The flat plate has a uniform thickness of 0.0125*c*, where in the literature thicknesses from 0 to 0.03*c* (Zuo et al., 2007; Meng and Sun, 2011) are used.

### Wing kinematics

The bee wing path is modeled as three angular velocities around the base of the wing. The rotational axis used in the present study are yaw (*θ*_1_), roll (*θ*_2_) and wing pitch (*θ*_3_). The angular velocities *θ*_1_, *θ*_2_ and *θ*_3_ are used to replicate the wing path during insect flight as a 2D simulation.

Kinematic data by Ellington and Dudley (Ellington, 1984; Ellington and Dudley, 1990) follow the path of a bee wingtip in forward flight at a variety of velocities using a high speed camera (Ellington and Dudley, 1990). The results are approximated as a change in magnitude of the *θ*_2_ component based on flight speed where *θ*_2*A*_ is equal to 10°, 15°, 20°, 24° and 26° for flight speeds from 1 to 5 m/s, respectively. The frequency *f* and angular amplitudes *θ*_1*A*_ and *θ*_3*A*_ are assumed constant at 150 Hz, 110° and 24°, respectively. The Reynolds number Re is used to nondimensionalize the flow, where Re=*ρ*(*V*_*kin*_+*V*_∞_)*c*/*μ*. Due to the nature of the problem, two different velocities are possible: the flow over the wing driven by the kinematics (*V_kin_*), and the flow over the wing by the freestream velocity (*V*_∞_). For the flight speeds under consideration, the Reynolds number varies from 987-1672, and is considered laminar flow.

The 3D angular amplitudes (*θ*_1*A*_, *θ*_2*A*_, *θ*_3*A*_) must be translated into a continuous two-dimensional motion. Of the three amplitudes, *θ*_3_ remains in angular form, as it is the angle of incidence of the wing. The approximated sinusoidal equation for the angular position is
(14)
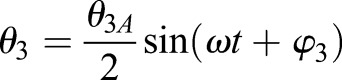
where *t* is the current time, *ω* is the angular frequency, and φ is the phase shift of the wing rotation based on the high speed videography performed by Ellington and Dudley (1990). The remaining two rotations, *θ*_1_ and *θ*_2_, are applied as translational motions in the *x*-and *y*-directions in 2D space. The translational equations are
(15)
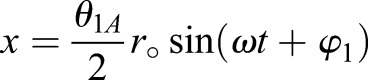

(16)
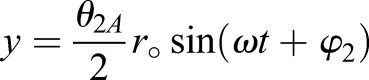
where *r*_°_ is the distance from the wing-base to the midpoint (*r*_°_=6.61 mm). The time derivatives of Eqns 14 and 15 are taken to determine the velocity of the wing relative to the bee in flight.

These 2D kinematic equations describe the representative up-and-down strokes shown in Fig. S2, moving from left to right. Starting from 0° angle of incidence (*τ*=0), the wing begins its upstroke, decreasing pitch angular velocity while increasing translational velocity until reaching 24° pitch at the upstroke midpoint (*τ*=0.25). For the latter half of the upstroke, the angular velocity increases while the translational velocity decreases until *θ*_3_ returns to 0°. At the top of the upstroke (*τ*=0.5) the wing translational velocities are zero, while the angular velocity is at the peak. The downstroke reverses the previous kinematics, returning to the original position at *τ*=1. The process is repeated for each beat of the wing.

### Domain dependency study

Solution dependency on computational domain size is explored for insect flight at a flight velocity of 5 m/s for domains of 14*c*×11*c*, 22*c* ×11*c* and 33*c*×11*c*. The bee wing cross-section is used for all analyses. Only the horizontal domain length is varied in the analysis. The vertical dimension remains constant because it was shown that a domain height of 10*c* was sufficient in previous studies (Kolomenskiy et al., 2011; Ishihara et al., 2014; Du and Sun, 2015). The instantaneous vorticity in the wing wake is shown in Fig. S3 and resulting aerodynamic forces (*C_L_* and *C_D_*) are shown in Fig. S4. Based on the aerodynamic forces and observed wake structures between the three computational domains, a domain of 22*c* ×11*c* is used for the analysis.

## Supplementary Material

Supplementary information

First Person interview
