# Supplementary material for: A computational study on the influence of insect wing geometry on bee flight mechanics
Source: Biol Open. 2017 Oct 23;6(12):1784–95. doi: 10.1242/bio.024612 (PMC5769640; doi:10.1242/bio.024612)
Supplement: Supplementary information [file biolopen-6-024612-s1.pdf]

© 2017. Published by The Company of Biologists Ltd | Biology Open (2017): doi:10.1242/bio.024612: Supplementary information

FIRST PERSON

First person – Jeffrey Feaster

First Person is a series of interviews with the first authors of a selection of papers published in Biology Open, helping early-career researchers promote themselves alongside their papers. Jeffrey Feaster is first author on 'A computational study on the influence of insect wing geometry on bee flight mechanics', published in BiO. Jeffrey is a research engineer in the labs of Francine Battaglia (CREST Lab) and Javid Bayandor (CRASH Lab) at the University at Buffalo, USA, investigating computational fluid dynamics and bio-inspired engineering systems.

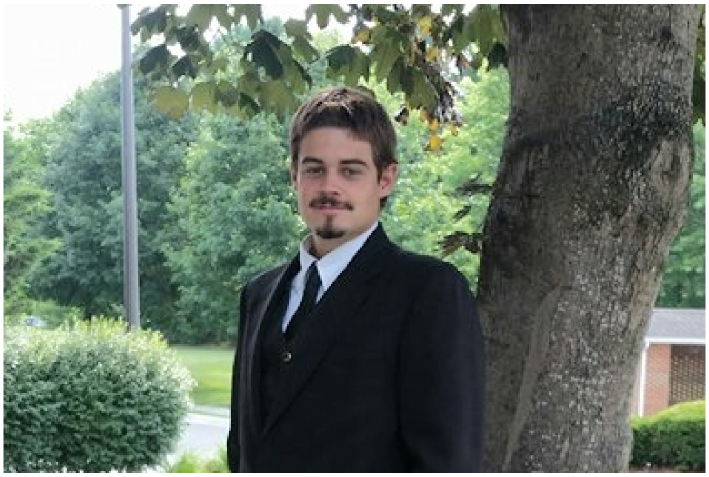

Jeffrey Feaster

What is your scientific background and the general focus of your lab?

My undergraduate and PhD degrees are both in mechanical engineering, focusing on fluids and thermodynamics. My research has focused on exploring and implementing dynamic modelling techniques to computational fluid dynamics analysis of non-terrestrial biological locomotion. My dissertation focused on applying dynamic modelling to bee flight to better understand the influences of wing morphology on aerodynamic performance. I am associated with two labs, the CRASH and CREST Labs. The CRASH Lab is a leader in computationally modelling highly complex fluid and structural phenomenon ranging from structural analysis of turbofan engines to fluid dynamic analysis of rajiform locomotion. The CREST Lab is a leader in computationally modelling building energy, combustion, multi-phase flows and renewable/alternative energy systems.

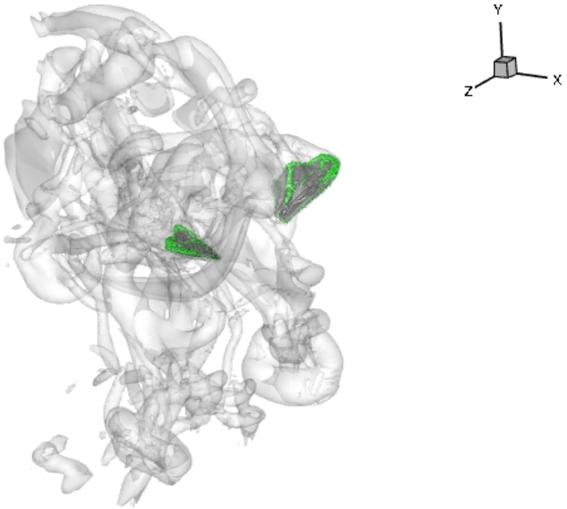

3D vortex structures from a bumblebee in forward flight.

How would you explain the main findings of your paper to non-scientific family and friends?

All of modern aerodynamics (fixed and rotor wing flight) stems from a geometry-centric approach to flight based on bird gliding where flow over a wing produces lift due to the wing's shape. Static flight has been a generally well-understood phenomenon for nearly a century, while insect flight is highly dynamic and has only been able to be explored in a quantitative manner for the past 30 years. In that time one of the major assumptions used in both computational and experimental analysis is that the wing cross-section can be extremely simplified. This paper aims to determine what aerodynamics have been overlooked by comparing the common cross-sections in the literature with a true cross-section from a bumblebee wing. Based on the findings presented in the paper, there can be significant differences in aerodynamic performance with cross-sectional variation. The vein structures of the biological model increase the frequency of leading-edge vortex shedding when compared to the simplified cross-section.

What are the potential implications of these results for your field of research?

Leading-edge vortex shedding can be part of a larger phenomenon known as dynamic stall, which can allow for a rapid increase in lift production, roughly 1.5 times the lift production of a static wing. The possibility of having a passive, geometric mechanism to regulate vortex shedding yields the opportunity to tailor vortex shedding and formation to application. Tailored vortex shedding, independent of wing kinematics, could allow for the optimisation of lift production, flight efficiency and overall stability.

What has surprised you the most while conducting your research?

Throughout my research, what has surprised me most is the inherent complexity of biological systems and the nuanced ways in which these systems come together to perform a particular function. For instance, some insect wing flapping frequencies can surpass the nerve

Jeffrey Feaster's contact details: Department of Mechanical and Aerospace Engineering, University at Buffalo, Buffalo, NY 14260, USA.

E-mail: jfeaster@buffalo.edu

Biology Open (2017): doi:10.1242/bio.024612: Supplementary information

impulse frequency, known as asynchronous muscles. Concerning the present paper, the fact that a bee wing with such an unintuitive wing geometry could have aerodynamic benefits came as a surprise.

What, in your opinion, are some of the greatest achievements in your field and how has this influenced your research?

The development of dynamic meshing techniques and the discovery and description of the leading edge vortices, trailing edge vortices, dynamic stall and clap-fling phenomenon have all been defining achievements for computational and experimental biological research. Dynamic meshing techniques facilitated an entire area of computational fluid dynamics focusing on highly complex phenomenon such as flying and swimming. Previous characterisation of such complex and dynamic phenomenon has inspired the present work to look deeper in an inquisitive and thorough fashion.

What changes do you think could improve the professional lives of early-career scientists?

A general push to attend more and different conferences and professional events, either intrinsically or by mentors/supervisors. I have had mentors who strongly believe in the importance of conference attendance and exposure, but I have had numerous colleagues who have not been so lucky. Exploration of different conferences and organizations can help early-career scientists and engineers to establish their ‘home conference’, a concept that often seems to go unmentioned during undergraduate and graduate school. Finding a conference setting where you regularly feel comfortable and have people willing to talk, interact, network, plan collaborations and feel present and heard is critical.

“Throughout my research, what has surprised me most is the inherent complexity of biological systems and the nuanced ways in which these systems come together to perform a particular function.”

What’s next for you?

Since completing my PhD, I have continued to work in computational fluid dynamics and am expanding my research interests to other forms of flying and swimming. In the future, I would like to move into industry pursuing a more research and design related career involving fluid dynamics.

Reference

Feaster, J., Battaglia, F. and Bayandor, J. (2017). A computational study on the influence of insect wing geometry on bee flight mechanics. *Biol. Open* **6**, doi:10.1242/bio.024612.
